# Sociocultural and Clinical Determinants of Sexual Dysfunction in Perimenopausal Women with and Without Breast Cancer

**DOI:** 10.3390/curroncol31110543

**Published:** 2024-11-20

**Authors:** Osiris G. Delgado-Enciso, Valery Melnikov, Gustavo A. Hernandez-Fuentes, Jessica C. Romero-Michel, Daniel A. Montes-Galindo, Veronica M. Guzmán-Sandoval, Josuel Delgado-Enciso, Mario Ramirez-Flores, Iram P. Rodriguez-Sanchez, Margarita L. Martinez-Fierro, Idalia Garza-Veloz, Karmina Sánchez-Meza, Carmen A. Sanchez-Ramirez, Carmen Meza-Robles, Ivan Delgado-Enciso

**Affiliations:** 1 Department of Molecular Medicine, School of Medicine, University of Colima, Colima 28040, Mexico; odelgado@ucol.mx (O.G.D.-E.); melnik@ucol.mx (V.M.); ghfuentes@ucol.mx (G.A.H.-F.); mario_ramirez@ucol.mx (M.R.-F.); ksmeza@ucol.mx (K.S.-M.); carmen_sanchez@ucol.mx (C.A.S.-R.); rmeza1@ucol.mx (C.M.-R.); 2 Faculty of Chemical Sciences, University of Colima, Coquimatlan 28400, Mexico; daniel_montes@ucol.mx; 3 Faculty of Law, University of Colima, Colima 28040, Mexico; jessica_romero@ucol.mx (J.C.R.-M.); derecho@ucol.mx (J.D.-E.); 4 School of Psychology, University of Colima, Colima 28040, Mexico; gus_vero@ucol.mx; 5 Foundation for Ethics, Education, and Cancer Research of the State Cancer Institute of Colima AC, Colima 28085, Mexico; 6 Molecular and Structural Physiology Laboratory, School of Biological Sciences, Universidad Autónoma de Nuevo León, San Nicolás de los Garza 66455, Mexico; iram.rodriguezsa@uanl.edu.mx; 7 Molecular Medicine Laboratory, Unidad Academica de Medicina Humana y Ciencias de la Salud, Universidad Autónoma de Zacatecas, Zacatecas 98160, Mexico; margaritamf@uaz.edu.mx (M.L.M.-F.); idaliagv@uaz.edu.mx (I.G.-V.); 8 State Cancerology Institute of Colima, Health Services of the Mexican Social Security Institute for Welfare (IMSS-BIENESTAR), Colima 28085, Mexico; 9 Robert Stempel College of Public Health and Social Work, Florida International University, Miami, FL 33199, USA

**Keywords:** breast cancer, cancer survivors, sexual life, psychological influences, anxiety, social development, sexual dysfunction

## Abstract

Breast cancer survivorship is a recognized risk factor for sexual dysfunction, with various clinical, sociocultural, and psychological factors potentially interacting differently across populations. This study compared sexual dysfunction, anxiety, and depression between females with breast cancer and those without, aiming to identify associated factors. A total of 362 females participated, including 227 with sexual dysfunction and 135 controls. Among them, 195 are breast cancer survivors, while 167 have no personal history of cancer. Key variables were analyzed using Student’s t-test for quantitative data and Fisher’s exact test for categorical data, while logistic regression models were used to assess the association between sexual dysfunction and various factors. Multivariate analysis revealed that, in sexually active females, breast cancer survivorship increased the odds of sexual dysfunction 2.7-fold (95% CI: 1.17–6.49; *p* = 0.020). Anxiety was significantly associated with sexual dysfunction, regardless of cancer status (AdOR 6.00; 95% CI: 2.50–14.43; *p* < 0.001). The interaction between cancer survival and anxiety further increased the odds of sexual dysfunction by more than 11-fold (AdOR 11.55; 95% CI: 3.81–35.04; *p* < 0.001). Additionally, obesity was found to be a protective factor among cancer survivors (AdOR 0.149; 95% CI: 0.027–0.819; *p* = 0.029). In conclusion, breast cancer has a significant impact on sexual function, with psychological factors like anxiety playing a crucial role. Addressing these issues requires a holistic, patient-centered approach that considers the complex interplay of physical, emotional, and sociocultural factors.

## 1. Introduction

Breast cancer remains one of the most prevalent malignancies affecting females worldwide, posing a significant public health concern due to its high incidence and impact on quality of life [1,2]. While advances in early detection and treatment have improved survival rates, survivors often face numerous long-term physical and psychological challenges, including sexual dysfunction, anxiety, depression, and body image issues [3].

In the international scenario, there is significant discourse on the challenges faced by breast cancer survivors, particularly regarding sexual dysfunction, anxiety, and depression, which affect females globally. Reports indicate that up to 50% of survivors in countries like Kenia [4] and Malaysia [5] experience sexual dysfunction, while cultural stigma in Japan exacerbates feelings of isolation and psychological distress [6]. Similarly, societal pressures in the Middle East [7] complicate emotional recovery, and nearly 30% of survivors in the United Kingdom face similar issues [8].

In the first place, sexual dysfunction is a common yet often overlooked consequence of breast cancer and its treatments, including chemotherapy, tamoxifen use, and surgical interventions. Research indicates that many females report significant psychological impacts stemming from these medical experiences, which can profoundly affect their sexual health and overall well-being. For instance, treatment-related changes may lead to fears about fertility loss, contributing to feelings of inadequacy and negative body image. Such concerns can manifest as feelings of sexual unattractiveness and a diminished sense of femininity, both of which are crucial aspects of a female’s identity. These psychological ramifications are not trivial; they can significantly impact mental health, leading to increased levels of anxiety and depression among survivors. Furthermore, the interplay of these factors often results in an altered sense of sexual identity, complicating the emotional landscape for females navigating life after breast cancer. Thus, it is imperative to recognize the multifaceted nature of these experiences to develop comprehensive support strategies that address both physical and psychological needs. These changes can lead to increased anxiety, depression, and an altered sense of sexual identity [9]. Sexual dysfunction significantly affects quality of life, with studies reporting that up to 83% of breast cancer survivors experience some form of sexual dysfunction—much higher than in the general population [3]. This dysfunction can affect various domains, including desire, arousal, lubrication, orgasm, satisfaction, and pain, commonly measured using tools like the Female Sexual Function Index (FSFI) [10,11].

Psychological factors such as anxiety and depression are also common among breast cancer survivors, with prevalence rates of 33.1% for anxiety and 18.2% for depression [12]. The diagnosis and treatment of cancer can be traumatic, increasing stress and mental health issues that may interfere with sexual functioning and overall well-being. Anxiety and depression not only reduce quality of life but also hinder recovery and post-treatment adjustment [13]. However, no studies have thoroughly examined whether breast cancer interacts with anxiety or depression to further increase the risk of sexual dysfunction. Understanding the relationship between psychological distress and sexual health is critical for developing comprehensive care plans that address both the physical and emotional needs of survivors.

Sociocultural factors, including education, socioeconomic status, religious affiliation, and lifestyle behaviors, significantly influence the health-related quality of life for females with breast cancer [12,14]. These factors directly impact sexual function, as higher educational attainment is associated with better health literacy and coping strategies, enabling females to navigate their treatment experiences more effectively. For instance, females with higher education levels may be better equipped to understand their diagnosis and engage in discussions about their sexual health, thereby reducing the negative effects of cancer diagnosis and treatment on their sexual well-being. Conversely, lower socioeconomic status can limit access to healthcare resources and support systems, exacerbating feelings of isolation and negatively affecting body image and sexual health. Additionally, the increased stress associated with financial constraints may contribute to worse health outcomes, further complicating the recovery process [3].

In Mexico, breast cancer is the most commonly diagnosed cancer among females, with rising incidence rates over recent decades [15]. Despite this, research on the sexual and psychological health of Mexican breast cancer survivors is limited. Cultural norms and stigma surrounding sexuality may further complicate the assessment and management of sexual dysfunction in this population [16]. Examining these issues within the Mexican context is essential to develop culturally sensitive interventions and healthcare policies. The health of Mexican females is not only important for Mexico but also for countries where Mexican immigrants reside, particularly the U.S. Mexican immigrants represent the largest group of Hispanic/Latino immigrants in the U.S., and their health has critical public health implications. Studies show that Mexican females, especially immigrants, are particularly vulnerable to mental health issues, highlighting the importance of understanding the influence of both nativity and gender on health outcomes [17].

This study aims to evaluate and compare the prevalence and associated factors of sexual dysfunction, breast cancer, and anxiety among Mexican females. As healthcare systems worldwide strive to improve the quality of life for breast cancer survivors, understanding the multifaceted challenges they face—particularly concerning sexual dysfunction and psychological distress—becomes increasingly essential. The findings from this study not only shed light on the specific experiences of Mexican females but also offer insights applicable to diverse populations, highlighting the universal need for comprehensive care that addresses both physical and emotional health. Understanding these associations will support the development of targeted strategies to improve the quality of life and holistic care for breast cancer survivors in Mexico.

## 2. Materials and Methods

### 2.1. Study Design and Participants

This observational, case-control study compared females with and without breast cancer. The study population comprised females aged 35–65 years attending the State Cancerology Institute of Colima, Mexico (IMSS-BIENESTAR) for breast cancer follow-up or preventive mammography. The hospital’s strategic location in Western Mexico, specifically serving the regions of Colima, Jalisco, and Michoacán, allows it to receive a diverse patient population from across the central-Pacific region. This influx brings together individuals from a range of cultural, religious, and socioeconomic backgrounds, creating a unique opportunity to study a variety of health-related behaviors and outcomes within this population. This study received ethical approval from the Research Ethics Committee of the State Cancerology Institute of Colima (approval number CEICANCL270418-VAGECANN-08, 11 March 2019). Informed consent was obtained from all participants, emphasizing the voluntary nature of the survey and ensuring confidentiality by not collecting any identifying information.

### 2.2. Variables and Measurement

The dependent variable was sexual dysfunction, evaluated through the Female Sexual Function Index (FSFI) [18,19]. The FSFI assesses sexual function across six domains: desire, arousal, lubrication, pain, orgasm, and satisfaction. It evaluates sexual function over the past 30 days and has been validated for use in females with breast cancer [10,11]. Due to concerns about the accuracy of FSFI scores in females who have not engaged in recent sexual activity—since 15 of the 19 FSFI items include a ‘0—No sexual activity’ category—the complete FSFI was only analyzed for sexually active females. However, the domains of desire, and especially satisfaction, allow for the evaluation of sexually inactive females. Scores of less than 3.6 points in each FSFI domain were considered indicative of a risk of sexual dysfunction in the assessed domain [20]. Based on the above, sexual dysfunction was defined as having an FSFI score of less than ≤21 in sexually active females, or a satisfaction domain score of less than 3.6 in sexually inactive females, as previously reported. [18,19]. Considering the above, participants were categorized into two groups: cases (females with sexual dysfunction) and controls (females without sexual dysfunction), including patients with and without breast cancer.

Independent variables included sociocultural and clinical factors. The main variable was a previous breast cancer diagnosis (yes/no). Other variables included age, marital status, education level, employment status, the use of breast prostheses, and the presence of comorbidities such as smoking (having smoked 100 or more cigarettes in their lifetime), diabetes, hypertension, and obesity. Anxiety and depression levels were measured using the Spanish version of the Hospital Anxiety and Depression Scale (HADS), which consists of two subscales: one for depression and one for anxiety. Clinical cut-off points were set at ≥11 to indicate probable moderate/severe anxiety or depression [21]. The presence of comorbidities was included because of their potential impact on physical and psychological well-being, which may influence sexual dysfunction. Factors such as diabetes, hypertension, obesity, and smoking are known to affect blood flow, hormonal balance, and stress levels, helping this study distinguish (in multivariate logistic regression analysis) the specific effects of breast cancer on sexual function from other chronic health-related influences [22,23].

### 2.3. Selection Criteria

The inclusion criteria for cases required participants to be females aged 35–65 years with a FSFI score ≤21 (in females sexually active in the last three months) or with a “satisfaction domain score” less than 3.6 in sexually inactive females, indicating sexual dysfunction. These females were either attending the State Cancerology Institute of Colima for breast cancer screening (with no cancer) or had a past diagnosis of breast cancer but were undergoing follow-up (not receiving oncological treatment for the past six months). To minimize selection bias, a consecutive sampling method was used, recruiting all eligible females who met the inclusion criteria during their visits to the State Cancerology Institute of Colima for either breast cancer screening or follow-up appointments. Recruitment occurred during the 2019–2020 period (before the mobility and isolation restrictions caused by the COVID-19 pandemic), ensuring a consistent and representative sample from the target population. Eligibility was confirmed through initial interviews and clinical assessments before enrollment. This approach allowed for a non-selective, ongoing recruitment process and, together with the use of validated assessment tools like the FSFI and HADS, helped enhance the internal validity of this study.

To be included in this study, the volunteers needed a Karnofsky Performance Scale score of 80% or higher, had to be born in Western Mexico (Colima, Jalisco, Michoacán), and had to provide informed consent. Inclusion criteria for controls mirrored those of the cases but without sexual dysfunction. Exclusion criteria for both groups included unwillingness to participate, lactation in the past six months, psychomotor deficits, cognitive impairments, current hospitalization, incomplete clinical history, current use of antidepressants, history of sexual assault, or diagnosed psychiatric disorders.

### 2.4. Sample Size Calculation

The sample size was calculated to determine whether being a cancer survivor is a risk factor for sexual dysfunction in sexually active females. Using probabilistic methods for a case-control study with a one-sided significance level of 0.95, a power of 0.8, a proportion of sexual dysfunction in females without cancer of 0.15, a case-to-control ratio of 3, and an odds ratio (OR) of 3.5 based on previous data [3], the required sample size was 132 subjects (99 controls and 33 cases) [24]. A one-sided calculation for sample size was selected as this directly aligns with the primary hypothesis of this study, which was that breast cancer survivors are at elevated risk for sexual dysfunction compared to females with no history of cancer (rather than testing for a bidirectional relationship). This approach maximizes the statistical power of this study while ensuring that the sample size remains efficient for detecting risk differences.

### 2.5. Statistical Analysis

Descriptive statistics were used to determine mean age, clinical stage percentages, and other relevant characteristics. Quantitative data were first tested for normal distribution using the Kolmogorov–Smirnov test. For data following a normal distribution, Student’s *t*-tests for independent samples were used to compare groups. Fisher’s exact test was employed to compare qualitative data (dichotomous variables) between cases and controls. To assess the association between sociocultural and clinical factors with sexual dysfunction, with breast cancer as an additional risk variable, multivariate binary logistic regression analyses were conducted to obtain adjusted odds ratios (AdORs) with 95% confidence intervals (CIs) and *p*-values. Variables for the multivariate model were selected using a backward stepwise selection method, with a threshold of 0.05 for entry and 0.10 for removal (only the final model is presented). A separate model was used to analyze the two-way interaction between breast cancer and anxiety. SPSS software version 20 (IBM, Armonk, NY, USA) was used for statistical analysis. The sample size was calculated using the Cleveland Clinic Department of Quantitative Health Sciences’ online sample size calculator [24]. The statistical power of the most relevant results was calculated using G*Power version 3.1.9.6 [25,26]. *p*-values less than 0.05 were considered statistically significant [27].

## 3. Results

### 3.1. General Overview and Group Comparison

**Figure 1:**
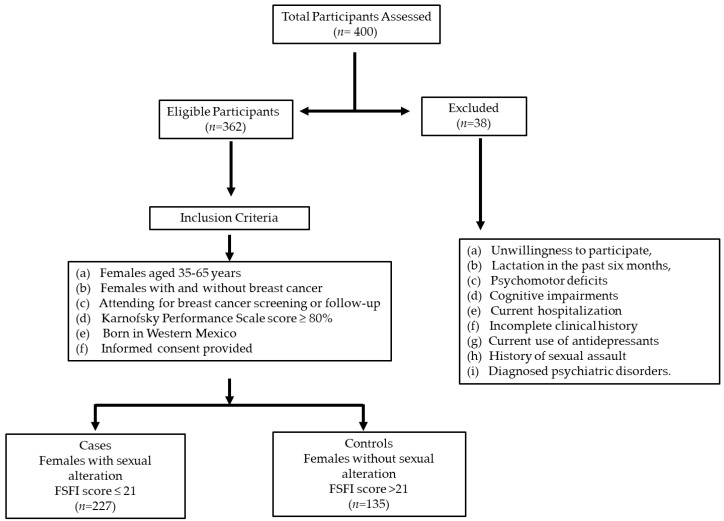
Flowchart of participant screening and inclusion for breast cancer study in Western Mexico.

A total of 362 females were included in this study, of which 227 and 135 were in the case and control groups, respectively (with and without sexual dysfunction) (Figure 1).

Table 1 shows the main clinical and social characteristics of the included patients, according to whether or not they have sexual dysfunction. The average age of the participants was 51.5 ± 9.6 years, finding that females with dysfunction are significantly older (48.6 ± 8.3 vs. 53.1 ± 9.9, *p* < 0.001) and have a lower Kasnosfky index (96.1 ± 5.6 vs. 93.3 ± 7.1, *p* < 0.001). In addition, females with sexual dysfunction have a higher proportion of menopause (54.8% vs. 72.2%, *p* = 0.001), anxiety (16.3% vs. 33.9%, *p* < 0.001), and depression (3.7% vs. 8.8%, *p* = 0.085). Sexually active females were in a much higher proportion in the group without sexual dysfunction than in the group of females with dysfunction (85.2% vs. 16.3%, *p* < 0.001). No differences were found in the proportion of females with breast cancer, diabetes, hypertension, obesity, alcoholism, education, among other variables (see Table 1).

In Table 2, the main characteristics of patients with and without cancer are compared. As shown, there were no significant differences between the age of the patients (*p* = 0.118). The Karnofsky index was lower in females with cancer (96.1 ± 5.4 vs. 92.7 ± 7.2, *p* < 0.001), although it is important to highlight that the averages in both groups were above 90, which indicates normal activity. It was also observed that there was a higher proportion of Catholics among the cancer patients (83.9% vs. 91.6%, *p* = 0.040) and a higher level of high school education or beyond (9.0% vs. 25.0%, *p* < 0.001) (see Table 1). It is observed that cancer patients had a later sexual debut (19.2 ± 4.5 vs. 20.3 ± 4.8, *p* = 0.037), had fewer children (3.4 ± 2.0 vs. 2.9 ± 2.4, *p* = 0.045), and had a higher proportion of menopause (55.0% vs. 82.6%, *p* < 0.001) compared to females without cancer. Interestingly, females with cancer had lower scores on the HADS anxiety (8.35 ± 4.5 vs. 7.3 ± 3.9, *p* = 0.029) and depression scales (5.3 ± 3.8 vs. 4.2 ± 3.4, *p* = 0.003) (see Table 2), although no significant differences were found in the proportion of females who scored 11 or higher (see Table 3).

Among sexually active females, the mean scores on the Female Sexual Function Index (FSFI) did not differ between groups (26.0 ± 4.9 vs. 24.6 ± 6.9, *p* = 0.169), nor in the domains examined (desire, arousal, lubrication, orgasm, or pain), except for the satisfaction domain, where cancer survivors had lower scores (4.5 ± 1.6) compared to the control group (4.9 ± 1.1, *p* = 0.042) (see Table 2). However, when the values were dichotomized (FSFI ≤ 21), sexual dysfunction was higher in cancer patients (15.2% vs. 31.4%, *p* = 0.016), as well as dysfunction in the arousal (31.8% vs. 52.3%, *p* = 0.009), orgasm (16.7% vs. 32.6%, *p* = 0.020), and satisfaction domains (10.6% vs. 25.6%, *p* = 0.015). Among sexually inactive females, the desire domain showed a higher proportion of females with dysfunction in the cancer group (85.1% vs. 93.6%, *p* = 0.038), but, interestingly, the proportion of females with anxiety (HADS-A > 11) was significantly higher in the without cancer group compared to the females under cancer follow-up (43.6% vs. 17.4%, *p* < 0.001) (see Table 3).

### 3.2. Factors Associated with Sexual Dysfunction

An analysis was conducted to identify factors associated with sexual dysfunction (FSFI ≤ 21) using a multivariate binary logistic regression to obtain adjusted odds ratios (AdORs). Only variables for the most parsimonious multivariate model, selected using the backward stepwise selection method, are shown in Table 4. Considering all females, factors associated with sexual dysfunction (FSFI ≤ 21 in sexually active females or with a “satisfaction domain score” less than 3.6 in sexually inactive females) were anxiety (AOR 3.4, 95% CI 1.9–6.2) and being 50 years or older (AOR 2.6, 95% CI 1.6–4.3), whereas having better functionality (higher Karnofsky index) was a protective factor (AOR 0.95, 95% CI 0.92–0.99). In sexually active females, being under follow-up for breast cancer increased the likelihood of sexual dysfunction by 2.7 times (95% CI 1.17–6.49), whereas anxiety was an even more significant factor, increasing this likelihood by 6 times (AdOR 6.0, 95% CI 2.5–14.4). Conversely, a high Karnofsky index was a protective factor (AdOR 0.97, 95% CI 0.96–0.98, *p* < 0.001). There was an interaction between being under cancer follow-up and having anxiety, significantly increasing the risk of sexual dysfunction (AdOR 11.5, 95% CI 3.81–35.04, *p* < 0.001). As shown in Table 4, being under cancer follow-up was also a risk factor for dysfunction in the domains of excitation, orgasm, and satisfaction (scores < 3.6), while anxiety was a risk factor across all domains. In sexually inactive females, only the domains of desire and satisfaction were evaluated. Notably, the main factors associated with sexual dysfunction in sexually active females, such as being under cancer follow-up or having anxiety, were not relevant in sexually inactive females. Interestingly, among sexually inactive females, being Catholic increased the risk of dysfunction in satisfaction (AdOR 2.9, 95% CI 1.1–7.9, *p* = 0.031).

### 3.3. Factors Associated with Sexual Dysfunction in Breast Cancer Survivors

Analyzing only the subgroup of patients with breast cancer who are sexually active, a multivariate analysis confirmed that anxiety is a risk factor, increasing the risk of sexual dysfunction by 19 times (AdOR 19.152, 95% CI 3.696–99.232, *p* < 0.001). In contrast, the presence of obesity was identified as a protective factor in this subgroup of patients (AdOR 0.149, 95% CI 0.027–0.819, *p* = 0.029). No other factors analyzed, including the presence of mastectomy, breast reconstruction, or family support (variables included in the analyses in Table 4), were significantly associated with sexual dysfunction.

Table 5 shows the proportion of sexually active females under follow-up for breast cancer with sexual dysfunction according to their clinical stage, whether they had a mastectomy, and the type of breast prosthesis used. Although sexual dysfunction is more frequent in females who had a mastectomy (16.7% vs. 33.8%) or in females without prostheses compared to those with breast implants (41.2% vs. 35.7%), no significant differences were found between these subgroups (*p* > 0.05 for all comparisons).

### 3.4. Anxiety, a Major Risk Factor: What Is It Associated with?

As anxiety is identified as the principal risk factor associated with sexual dysfunction, it is pertinent to explore which variables are also associated with this disorder in sexually active females. Consistent with previous findings, sexual dysfunction itself is associated with a higher risk of anxiety (AdOR 8.801, 95% CI 2.933–26.413). Other significant risk factors include alcoholism (AdOR 4.112, 95% CI 1.166–14.495) and being of medium/high socioeconomic status (AdOR 9.831, 95% CI 1.086–88.946). On the other hand, a high Karnofsky index (AdOR 0.905, 95% CI 0.841–0.973) and having personal income (AdOR 0.191, 95% CI 0.053–0.689) are protective factors (see Table 6).

### 3.5. Statistical Power of Results

To ensure the validity of the results, statistical power was calculated for the most relevant findings using the G*Power software version 3.1.9.6 [25,26]. The statistical test used was “Exact—Proportions: Inequality, two independent groups (unconditional),” and the analysis was “Post hoc: Compute achieved power.” Calculations were performed with α = 0.05, one-tailed, using the pertinent data for each group and their respective adjusted odds ratio (AdOR) [27,28,29]. For sexually active females, the power values for the association between sexual dysfunction (FSFI ≤ 21) and breast cancer or anxiety were high, at 0.83 and 0.99, respectively. For sexually inactive females, despite the significant association between dysfunction in satisfaction domain and identifying as Catholic, the achieved power was medium (0.67). In the analysis including only breast cancer patients, the power values for associations between sexual dysfunction (FSFI ≤ 21) and anxiety or obesity were high, at 0.99 and 0.81, respectively.

Comparisons of the proportion of sexual dysfunction with or without mastectomy or implant had power values of 0.31 and 0.08, respectively, indicating that these results should be considered descriptive, as there was inadequate validity for comparing groups without significant differences. In the analysis of sexually active females to determine the association between anxiety and factors such as sexual dysfunction, alcoholism, and having personal income, the power values were high, at 0.99, 0.89, and 0.80, respectively. However, the association between anxiety and medium/high socioeconomic status had a power value of 0.59, suggesting it cannot be considered a definitive result.

## 4. Discussion

The present study provides valuable insights into the multifaceted impact of breast cancer and psychological health on sexual function among Mexican females. Breast cancer survivors exhibit a significantly higher prevalence of sexual dysfunction compared to females without cancer, particularly in the domains of excitation, orgasm, and satisfaction. A multivariate analysis showed that being a breast cancer survivor increased the odds of sexual dysfunction 2.7-fold (95% CI: 1.17–6.49; *p* = 0.020) in sexually active females. Furthermore, regardless of cancer status, anxiety emerged as a prominent factor associated with sexual dysfunction (AdOR 6.00; 95% CI: 2.50–14.43; *p* < 0.001). An interaction between being a cancer survivor and having anxiety was also demonstrated, which highly significantly increased the odds of sexual dysfunction by more than 11-fold (AdOR 11.55; 95% CI: 3.81–35.04; *p* < 0.001), highlighting the intricate interplay between psychological distress and sexual health in breast cancer survivors.

Consistent with the previous literature, breast cancer survivors were 2 to 2.7 times more likely to report sexual problems [30,31]. The increased rates of sexual dysfunction observed among survivors may be attributed to various treatment-related factors such as hormonal changes induced by chemotherapy and radiotherapy, physical alterations from surgical interventions like mastectomy, and resultant body image disturbances [3,31]. These physical sequelae can diminish sexual desire, impair arousal, and reduce overall sexual satisfaction, underscoring the need for integrative care approaches that address both medical and psychosocial aspects of survivorship.

Anxiety was identified as a significant predictor of sexual dysfunction across multiple domains among sexually active females, with those experiencing anxiety having up to six times higher odds of reporting sexual difficulties. Epidemiological studies have identified anxiety disorders as risk factors for reduced sexual desire and arousal, with recent research also finding strong associations between anxiety and orgasmic difficulties, as well as sexual pain. The heightened activity of the sympathetic nervous system during sexual arousal, which increases genital congestion, may involve non-genital sensations that an anxious female might perceive as threatening, thus diminishing the potential for sexual pleasure [3,32]. Mental health problems are among the most important risk factors for female sexual dysfunction. Despite frequent difficulties with sexuality, the importance of sexuality to females with mental illnesses cannot be overstated [3,33]. The heightened anxiety levels may stem from fears of cancer recurrence, uncertainties about future health, and coping with the demands of ongoing medical surveillance [34]. Although sexual dysfunction and anxiety have been shown to be frequent aspects in breast cancer survivors, the interaction between these factors had not been demonstrated previously, making this one of the main findings of the present study [3,13,33]. The interaction between breast cancer status and anxiety notably amplified the risk of sexual dysfunction, suggesting that these factors may synergistically exacerbate sexual health challenges in survivors.

Interestingly, although females without cancer exhibited higher overall anxiety scores, the prevalence of clinically significant anxiety (HADS-A ≥ 11) was found to be greater in females without cancer, with rates of 22.1% for those with cancer compared to 33.5% for those with cancer (*p* = 0.013, see Table 2). This finding contrasts with previous research indicating that anxiety prevalence is generally higher in cancer survivors than in healthy controls (17.9% vs. 13.9%, *p* = 0.0039). The differences observed in our study may stem from various factors, such as the specific characteristics of our patient population or the methodologies employed in previous studies. Nevertheless, among sexually active females, the prevalence of anxiety was found to be greater in cancer survivors than in their non-cancer counterparts (see Table 3), aligning with findings from previous studies [35]. Further research is necessary to investigate these dynamics and to assess the longitudinal trajectories of psychological distress in breast cancer populations.

The Karnofsky Performance Status (KPS) index emerged as a protective factor against sexual dysfunction in sexually active females, indicating that better functional status correlates with improved sexual health (r = 0.163, *p* = 0.045). This association underscores the importance of physical rehabilitation and maintenance of functional abilities in enhancing quality of life and sexual well-being among survivors [36]. Interventions aimed at improving physical fitness and managing treatment-related side effects could thus play a critical role in mitigating sexual difficulties [36].

Sociodemographic factors also influenced sexual function outcomes. Higher educational attainment was more prevalent among breast cancer survivors and may contribute to increased health literacy, better access to healthcare resources, and more effective utilization of supportive services [3]. However, this study did not find significant associations between socioeconomic status or employment and sexual dysfunction, suggesting that other factors may have more substantial impacts in this context. Interestingly, alcoholism was found to be a protective factor for dysfunction in the arousal domain (AdOR 0.318, 95% CI 0.110–0.921). A previous study revealed that beliefs that drinking enhances and disinhibits sexual experience are widely accepted, and that those with strong expectations of sexual enhancement drink significantly more on sexual occasions than those without. However, only those with strong expectations of enhancing their sexual function by drinking alcohol reported greater arousal at high levels of consumption, while those with weak expectations of enhancement reported lower arousal [37]. This is consistent with the results of the present study, where drinking alcohol protected against dysfunction precisely in the arousal domain. However, it should be noted that, based on previous reports, this finding is not generalized to all subjects, and that alcoholism in our study was a risk factor with the presence of anxiety. The protective effect observed may be confounded by other lifestyle or psychosocial factors and should be interpreted cautiously. For the above reasons, further studies should be conducted in the general population and in the context of female cancer survivors.

The choice to focus on Catholicism in this study was driven by its high prevalence in the region, which may influence the observed outcomes [38]. Religious affiliation, particularly Catholicism, was associated with increased dysfunction in sexual satisfaction among sexually inactive females. This finding may reflect cultural and religious beliefs influencing attitudes toward sexuality [39], potentially leading to feelings of guilt, shame, or restricted sexual expression. Culturally sensitive counseling and education could help address these issues by promoting open dialogue and challenging detrimental stereotypes surrounding sexuality and illness. It would be important to explore the impact of other religious affiliations and belief systems, as different perspectives may influence sexual health and well-being in various ways. Additionally, examining how alternative medicines, such as herbal remedies, might intersect with these beliefs and affect sexual health is crucial [40].

The finding that obesity was identified as a protective factor against sexual dysfunction in breast cancer survivors is unexpected, as obesity is typically associated with an increased risk of various health issues, including sexual dysfunction [41]. However, this result may reflect complex biological, psychological, and social factors. From a biological perspective, obesity may lead to higher estrogen levels due to the peripheral conversion of androgens in adipose tissue. These elevated estrogen levels could have a protective effect on sexual function in females who have undergone treatments that drastically reduce hormone levels, such as chemotherapy or tamoxifen therapy [42]. Psychologically, some females with obesity might develop greater self-acceptance or a reduced concern about body image following cancer treatment, potentially lowering anxiety related to sexual activity and thereby improving sexual function. The psychological effect of body weight and body image and their probable influence on sexual function is influenced by cultural factors. An earlier study involving Mexican American females found that those with a stronger orientation towards Anglo culture displayed a greater preference for slimmer body types. In contrast, obese females, compared to those of normal weight, selected larger figures as their ideal, realistic, and attractive body types [43]. This demonstrates that culture influences body image and size perceptions [43,44,45]; the result is consistent with previous studies that show that a high body mass index is associated with improved sexual functioning in endometrial, ovarian, and vulvar cancer patients [46] but not in patients with cervical cancer. Although sexual dysfunction and anxiety are frequently observed in breast cancer survivors, the interaction between these factors, which notably amplified the risk of sexual dysfunction, is an important finding of the present study. This suggests that breast cancer status and anxiety may synergistically exacerbate sexual health challenges in survivors [3,13]. The interaction between breast cancer status and anxiety notably amplified the risk of sexual dysfunction, suggesting that these factors may synergistically exacerbate sexual health challenges in survivors but need a deeper exploration.

This study’s limitations include its case-control design, which restricts the ability to establish causality or assess temporal relationships between breast cancer, anxiety, and sexual dysfunction. The reliance on self-reported measures may also introduce reporting bias. As to the sample size, although adequate for most analyses, in some association results, confidence intervals may have been wide, which could indicate a small sample size for the analysis. Therefore, future studies with a larger sample size are recommended. The sample used also limits the generalizability of some findings, particularly in subgroup analyses like sexual orientation in the data collection process. Sexual orientation can play a significant role in shaping the experiences of breast cancer survivors, influencing factors such as support systems, relationship dynamics, and perceptions of body image. By not accounting for this aspect, this study may not fully capture the diverse challenges faced by individuals from different sexual orientations. Future research should prioritize the inclusion of sexual orientation data to ensure a more comprehensive understanding of the unique experiences and needs of all breast cancer survivors. Additionally, this study did not account for all potential confounders, such as medication effects or detailed treatment history, which could impact sexual health outcomes. Future studies with larger, longitudinal samples and more comprehensive data collection are needed to validate these findings and explore the mechanisms underlying the observed associations.

A valuable perspective for future research would be to expand the study’s geographic scope to include participants from the northern and southern regions of Mexico. This broader inclusion would allow for comparisons and richer analyses of findings across diverse cultural and lifestyle backgrounds, strengthening the applicability of the results. By encompassing a wider range of regional influences, such research could offer a more comprehensive understanding of the factors affecting the study outcomes and enhance the generalizability of the conclusions across different parts of the country.

Nevertheless, this study offers several strengths. It provides critical insights into the sexual and psychological health of breast cancer survivors within a cultural context that has been underrepresented in existing research. Given the significant issues related to gender differences and the entrenched “machismo” in this region [47], this study represents a notable advancement in addressing sexual dysfunction among survivors. By focusing on this specific population, the research underscores the importance of culturally sensitive and personalized approaches to managing sexual dysfunction. Incorporating evidence-based interventions, such as cognitive-behavioral therapy and targeted anxiety management counseling, could greatly enhance sexual health and emotional well-being. Moreover, educational programs for both patients and healthcare providers about sexuality and mental health could help mitigate stigma and encourage open communication. Continuous professional training is essential to meet the complex needs of survivors effectively. Additionally, future studies should consider including the perspectives of sexual partners, as understanding their experiences and challenges can provide a more comprehensive view of the impact of sexual dysfunction and contribute to more effective support strategies. Longitudinal and intervention studies are recommended to assess the effectiveness of these strategies across various cultural and clinical contexts, aiming to develop care models adapted to the diverse needs of patients.

## 5. Conclusions

This study highlights the significant impact of breast cancer and anxiety on sexual function in Mexican females. Breast cancer survivors experience higher rates of sexual dysfunction, particularly in the excitation, orgasm, and satisfaction domains. Anxiety exacerbates these issues, with a notable interaction effect in cancer survivors. Addressing both the physical and psychological aspects of sexual health is crucial for improving quality of life in breast cancer survivors. Integrating psychological support, addressing cultural and religious influences, and considering the complex role of obesity in sexual health are essential components of comprehensive care for females affected by breast cancer.

## Figures and Tables

**Table 1:** Main clinical characteristics of females included in this study according to their sexual dysfunction status.

Characteristics	All	Sexual Dysfunction		*p*
	(*n* = 362)	No (*n* = 135)	Yes (*n* = 227)	
Age	51.54 ± 9.6	48.6 ± 8.3	53.2 ± 9.9	<0.001
Breast cancer	53.9% (195)	51.9% (70)	55.1% (125)	0.586
Diabetes	22.4% (81)	17.0% (23)	25.6% (58)	0.068
Hypertension	30.9% (112)	28.9% (39)	32.1% (73)	0.558
Obesity	43.1% (156)	45.2% (61)	41.9% (95)	0.584
Alcoholism	15.2% (55)	15.6% (21)	15.0% (34)	0.881
Smoking	16.6% (60)	17. 0% (23)	16.6% (37)	0.884
Drug use	2.8% (10)	2.2% (3)	3.1% (7)	0.750
Karnofsky index	94.2 ± 6.6	96.1 ± 5.6	93.3 ± 7.1	<0.001
Catholic	88.4% (320)	91.1% (123)	86.8% (197)	0.239
High school or higher	17.7% (64)	16.3% (22)	18.5% (42)	0.670
Occupation ^α^				0.136
Homemaker	63.3% (229)	65.2% (88)	62.1% (141)	
Unemployed	7.7% (28)	4.4% (6)	9.7% (22)	
Employed	28.5% (103)	30.4% (41)	27.3% (62)	
Retired	0.60% (2)	0.0% (0)	0.9% (2)	
Own income	30.1% (109)	31.1% (42)	29.5% (67)	0.813
Low socioeconomic level	19.9% (72)	19.3% (26)	20.3% (46)	0.892
Age at first sexual intercourse (IVSA)	19.9 ± 4.7	20.1 ± 4.7	19.6 ± 4.7	0.537
Contraceptive method ^β^				0.238
None	45.0% (163)	37.0% (50)	49.8% (113)	
IUD	15.7% (57)	21.5% (29)	12.3% (28)	
Hormonal ^∞^	24.5% (89)	27.4% (37)	22.9% (52)	
Surgical	11.9% (43)	11.8% (16)	11.9% (27)	
Barrier	2.2% (8)	2.2% (3)	2.2% (5)	
Natural	0.5% (2)	0.0% (0)	0.9% (2)	
Breastfeeding	88.9% (322)	89.6% (121)	88.5% (201)	0.723
Number of children	3.2 ± 2.3	3.2 ± 2.3	3.2 ± 2.6	0.907
Pregnancies	91.4% (331)	94.8% (128)	89.4% (203)	0.132
Menopause	65.7% (238)	54.8% (74)	72.2% (164)	0.001
HADS anxiety score	7.8 ± 4.3	6.9 ± 3.8	8.3 ± 4.5	0.003
HADS depression score	4.8 ± 3.7	4.1 ± 3.3	5.1 ± 3.8	0.010
Anxiety	27.3% (99)	16.3% (22)	33.9% (77)	<0.001
Depression	6.9% (25)	3.7% (5)	8.8% (20)	0.085
Active sexual life	42.0% (152)	85.2% (115)	16.3% (37)	<0.001

Sexual Dysfunction: Female Sexual Function Index score ≤21 in sexually active females or a “satisfaction domain score” less than 3.6 (satisfaction dysfunction) in sexually inactive females. HADS: Hospital Anxiety and Depression Scale, with a score of 0 to 21 for depressive symptoms and 0 to 21 for anxiety. Clinical cut-off points were set at ≥11 for the presence of anxiety or depression. ^∞^ Use of sex hormones for contraceptive purposes or as replacement therapy. Comparisons were made using the Student’s *t*-test for independent samples (quantitative data) or Fisher’s exact test (for qualitative, dichotomous, or polytomous data). ^α^ Fisher exact test using 2 × 4 contingency table to observe if the occupation of the participants (four categories) is different between those who have vs. those who do not have sexual dysfunction. ^β^ Fisher exact test using 2 × 6 contingency table to observe if the contraceptive method of the participants (six categories) is different between those who have vs. those who do not have sexual dysfunction.

**Table 2:** Main clinical and reproductive characteristics, as well as scores on depression, anxiety, and sexual function instruments, according to breast cancer status.

Characteristics	All	Breast Cancer		*p*
	(*n* = 362)	No (*n* = 167)	Yes (*n* = 195)	
Age	51.54 ± 9.67	50.68 ± 10.31	52.27 ± 9.05	0.118
Diabetes	22.4% (81)	21.5% (36)	23.1% (45)	0.801
Hypertension	30.9% (112)	29.3% (49)	32.3% (63)	0.571
Obesity	43.1% (156)	49.1% (82)	38.4% (75)	0.044
Alcoholism	15.2% (55)	20.1% (35)	10.3% (20)	0.005
Smoking	16.6% (60)	18.0% (30)	15.4% (30)	0.571
Drug use	2.8% (10)	1.8% (3)	3.6% (7)	0.1970
Karnofsky index	94.2 ± 6.6	96.1 ± 5.5	92.7 ± 7.2	<0.001
Catholic	88.4% (320)	83.8% (140)	92.3% (180)	0.013
High school or higher	17.7% (64)	9.0% (15)	25.1% (49)	<0.001
Occupation ^α^				0.624
Homemaker	63.3% (229)	59.9% (100)	66.1% (129)	
Unemployed	7.7% (28)	7.2% (12)	8.2% (16)	
Employed	28.5% (103)	31.7% (53)	25.6% (50)	
Retired	0.6% (2)	06% (1)	0.5% (1)	
Own income	30.1% (109)	32.3% (54)	28.2% (55)	0.422
Low socioeconomic level	19.9% (72)	15.0% (25)	24.1% (47)	0.026
Age at first sexual intercourse (IVSA)	19.9 ± 4.7	19.3 ± 4.7	20.4 ± 4.7	0.030
Contraceptive method ^β^				0.062
None	45.0% (163)	49.7% (83)	41.0% (80)	
IUD	15.7% (57)	20.4% (34)	11.8% (23)	
Hormonal ^∞^	24.5% (89)	23.9% (40)	25.1% (49)	
Surgical	11.9% (43)	7.2% (12)	15.9% (31)	
Barrier	2.2% (8)	2.4% (4)	2.1% (4)	
Natural	0.5% (2)	0.0% (0)	0.9% (2)	
Breastfeeding	88.9% (322)	89.8% (150)	88.2% (172)	0.862
Number of children	3.2 ± 2.3	3.5 ± 2.1	2.9 ± 2.4	0.045
Pregnancies	91.4% (331)	95.8% (160)	87.7% (171)	0.015
Menopause	65.7% (238)	49.7% (83)	77.9% (155)	<0.001
Previous mammogram	74.8% (271)	53.7% (90)	94.9% (185)	<0.001
HADS anxiety score	7.8 ± 4.3	8.3 ± 4.6	7.3 ± 3.9	0.029
HADS depression score	4.8 ± 3.7	5.4 ± 3.8	4.2 ± 3.4	0.003
Anxiety	27.3% (99)	33.5% (56)	22.1% (43)	0.013
Depression	6.9% (25)	9.6% (16)	4.6% (9)	0.094
Active Sexual Life	42.0% (152)	39.5% (66)	44.1% (86)	0394
FSFI in sexually active females				
Desire	3.21 ± 1.17	3.30 ± 0.99	3.15 ± 1.29	0.425
Arousal	3.62 ± 1.24	3.81 ± 0.99	3.47 ± 1.39	0.079
Lubrication	4.45 ± 1.34	4.58 ± 1.21	4.36 ± 1.44	0.325
Orgasm	4.59 ± 2.91	5.03 ± 4.02	4.25 ± 1.55	0.105
Satisfaction	4.73 ± 1.43	4.98 ± 1.11	4.53 ± 1.61	0.042
Pain	4.87 ± 1.28	4.86 ± 1.30	4.87 ± 1.28	0.931
FSFI Total	25.30 ± 6.17	26.08 ± 4.97	24.69 ± 6.92	0.169
FSFI domains in sexually inactive females				
Desire	1.81 ± 0.87	2.02 ± 0.96	1.62 ± 0.73	0.001
Satisfaction	2.2 ± 0.91	2.19 ± 0.91	2.13 ± 0.91	0.678

FSFI: Female Sexual Function Index. HADS: Hospital Anxiety and Depression Scale, with a score of 0 to 21 for depressive symptoms and 0 to 21 for anxiety. Clinical cut-off points were set at ≥11 for the presence of anxiety or depression. ^∞^ Use of sex hormones for contraceptive purposes or as replacement therapy. Comparisons were made using the Student’s *t*-test for independent samples (quantitative data) or Fisher’s exact test (for qualitative, dichotomous, or polytomous data). ^α^ Fisher exact test using 2 × 4 contingency table to observe if the occupation of the participants (four categories) is different between those who have vs. those who do not have breast cancer. ^β^ Fisher exact test using 2 × 6 contingency table to observe if the contraceptive method of the participants (six categories) is different between those who have vs. those who do not have breast cancer.

**Table 3:** Frequency of sexual dysfunction in various domains according to whether or not they have breast cancer.

Sexual Dysfunction *		Cancer		
	All	No	Yes	*p*
In sexually active female	*n* = 152	*n* = 66	*n* = 86	
Dysfunction				
General ≤ 21	24.3% (37)	15.2% (10)	31.4% (27)	0.016
Desire	48.0% (73)	47.0% (31)	48.8% (42)	0.474
Arousal	43.4% (66)	31.8% (21)	52.3% (45)	0.009
Lubrication	23.0% (35)	18.2% (12)	26.7% (23)	0.147
Orgasm	25.7% (39)	16.7% (11)	32.6% (28)	0.020
Satisfaction	19.1% (29)	10.6% (7)	25.6% (22)	0.015
Pain	12.5% (19)	15.2% (10)	10.5% (9)	0.267
HADS				
Anxiety ≥ 11	23.7% (36)	19.7% (13)	26.7% (23)	0.207
Depression ≥11	3.9% (6)	6.1% (4)	2.3% (2)	0.225
In sexually inactive female	*n* = 210	*n* = 101	*n* = 109	
Dysfunction				
Desire	89.5% (188)	85.1% (86)	93.6% (102)	0.038
Satisfaction	87.1% (183)	85.1% (86)	89.0% (97)	0.266
HADS				
Anxiety ≥11	30.0% (63)	43.6% (44)	17.4% (19)	<0.001
Depression ≥ 11	9.0% (19)	11.9% (12)	6.4% (7)	0.128

*General sexual dysfunction (FSFI index ≤ 21) and all domains (domain with score < 3.6) were calculated only for sexually active females at the time of the interview. For sexually inactive females, only desire and satisfaction domains were calculated. Anxiety (≥11 in the anxiety subscale of HADS); depression (≥11 in the depression subscale of HADS). Comparisons were made using Fisher’s exact test.

**Table 4:** Multivariate logistic regression to detect factors associated with sexual dysfunction overall and in different domains.

Variable	Ad OR	95% CI		*p*
All Females (*n* = 362)				
≥50 years old	2.688	1.668	4.332	0.020
Karnofsky Index	0.957	0.922	0.994	<0.001
Anxiety	3.452	1.900	6.274	<0.001
Sexually Active Females (*n* = 152)				
Factors Associated with Sexual Dysfunction (FSFI < 21)				
Cancer	2.764	1.176	6.494	0.020
Anxiety	6.008	2.500	14.435	<0.001
Karnofsky Index	0.977	0.969	0.985	<0.001
Interaction Cancer * Anxiety with Sexual Dysfunction (FSFI < 21)				
Cancer * Anxiety	11.550	3.818	35.045	<0.001
Factors Associated with Dysfunction in the Desire Domain				
Anxiety	2.200	1.042	4.646	0.039
Factors Associated with Dysfunction in the Excitation Domain				
Cancer	2.780	1.391	5.555	0.004
Anxiety	3.722	1.524	9.091	0.004
Alcoholism	0.318	0.110	0.921	0.035
IVSA > 18	0.307	0.163	0.579	<0.001
Factors Associated with Dysfunction in the Lubrication Domain				
Anxiety	4.139	1.783	9.609	0.001
Karnofsky Index	0.983	0.978	0.988	<0.001
Factors Associated with Dysfunction in the Orgasm Domain				
Cancer	2.487	1.081	5.721	0.032
Anxiety	5.653	2.379	13.432	<0.001
Karnofsky Index	0.979	0.971	0.986	<0.001
Factors Associated with Dysfunction in the Satisfaction Domain				
Cancer	2.875	1.126	7.341	0.027
Anxiety	5.622	2.270	13.928	<0.001
Karnofsky Index	0.973	0.965	0.982	<0.001
Factors Associated with Dysfunction in the Pain Domain				
Anxiety	3.757	1.388	10.171	0.009
Karnofsky Index	0.976	0.969	0.982	<0.001
Sexually Inactive Females (*n* = 210)				
Factors Associated with Dysfunction in the Desire Domain				
No Associated Factors				
Factors Associated with Dysfunction in the Satisfaction Domain				
Catholic	2.963	1.103	7.961	0.031
Karnofsky Index	1.007	0.998	1.017	0.107

A multivariate binary logistic regression analysis was performed to obtain adjusted odds ratios (AdORs) with 95% confidence intervals (CIs) and *p*-values. Variables for the most parsimonious multivariate model were selected using the backward stepwise selection method. The entry probability for the stepwise regression was set at 0.05 and the removal probability at 0.10 (only the final step is shown). *The variables included in the model were those that differed between patients with and without cancer (as shown in Table 1) and other relevant variables such as age and hormone use, age 50 or older, Karnofsky index, presence of breast cancer, anxiety (≥11 on the anxiety subscale of the HADS), depression (≥11 on the depression subscale of the HADS), Catholic religion, education at high-school level or higher, alcoholism, obesity, menopause, initiation of sexual activity at age 18 or older (IVSA ≥ 18), and use of hormonal contraceptives or hormone replacement therapy.

**Table 5:** Sexual dysfunction (FSFI ≤ 21) by clinical stage, mastectomy status, and type of breast prosthesis in sexually active females.

Factor	Sexual Dysfunction (FSFI ≤ 21)			*p*
		No	Yes	
Clinical Stage				0.300
I-II	*n* = 62 (100%)	66.1%	33.9%	
III-IV	*n* = 24 (100%)	75.0%	25.0%	
Mastectomy				0.201
No	*n* = 12 (100%)	83.3%	16.7%	
Yes	*n* = 74 (100%)	66.2%	33.8%	
Type of Breast Prosthesis *				0.604
None	*n* = 17 (100%)	58.8%	41.2%	
Trapo ^a^	*n* = 12 (100%)	75.0%	25.0%	
Seed bag ^b^	*n* = 3 (100%)	100%	0.0%	
Gel ^c^	*n* = 24 (100%)	62.5%	37.5%	
Colgajo ^d^	*n* = 4 (100%)	75.0%	25.0%	
Implant	*n* = 14 (100%)	64.3%	35.7%	

* Only in patients with mastectomy (*n* = 74). Comparisons were performed using Fisher’s exact test (for dichotomous or polytomous data). ^a^ Trapo: a cloth or fabric pad used as a type of prosthesis. ^b^ Seed bag: a bag filled with birdseed, scientifically known as Canary seed. ^c^ Gel: a silicone gel prosthesis used for breast reconstruction. ^d^ “Colgajo”: a flap, a surgical reconstruction technique using a flap of tissue from another part of the body to reconstruct the breast.

**Table 6:** Multivariate logistic regression to detect factors associated with anxiety.

Variable	Ad OR	95% CI		*p*
Sexual Dysfunction	8.801	2.933	26.413	<0.001
Karnofsky Index	0.905	0.841	0.973	0.007
Alcoholism	4.112	1.166	14.495	0.028
Medium/High Socioeconomic Status	9.831	1.086	88.946	0.042
Personal Income	0.181	0.053	0.689	0.011

Sexual Dysfunction: Female Sexual Function Index ≤ 21. Medium/High Socioeconomic Status: medium/high socioeconomic level. Personal Income: receives remuneration from any formal or informal activity performed. A multivariate binary logistic regression analysis was conducted to obtain adjusted odds ratios (AdORs) with 95% confidence intervals (CIs) and *p*-values. The most parsimonious multivariate model was selected using a backward stepwise selection method. The probability for variable entry into the model was set at 0.5, and the probability for variable removal was set at 0.10 (only the final step is shown). Variables included in the model were breast cancer presence, age ≥ 50 years, Karnofsky index, depression (≥11 on the HADS depression subscale), Catholic religion, education level ≥high school, alcoholism, diabetes, hypertension, lactation, obesity, menopause, sexual initiation at ≥18 years (IVSA ≥18), and hormonal use for contraception or hormone replacement therapy.

## Data Availability

The datasets used and/or analyzed during the current study are available from the corresponding author upon reasonable request.

## References

[1] Li Q., Xia C., Li H., Yan X., Yang F., Cao M., Zhang S., Teng Y., He S., Cao M. (2024). Disparities in 36 Cancers across 185 Countries: Secondary Analysis of Global Cancer Statistics. Front. Med..

[2] Schumacher A.E., Kyu H.H., Aali A., Abbafati C., Abbas J., Abbasgholizadeh R., Abbasi M.A., Abbasian M., Abd ElHafeez S., Abdelmasseh M. (2024). Global Age-Sex-Specific Mortality, Life Expectancy, and Population Estimates in 204 Countries and Territories and 811 Subnational Locations, 1950–2021, and the Impact of the COVID-19 Pandemic: A Comprehensive Demographic Analysis for the Global Burden of Disease Study 2021. Lancet.

[3] Sousa Rodrigues Guedes T., Barbosa Otoni Gonçalves Guedes M., de Castro Santana R., Costa da Silva J.F., Almeida Gomes Dantas A., Ochandorena-Acha M., Terradas-Monllor M., Jerez-Roig J., Bezerra de Souza D.L. (2022). Sexual Dysfunction in Women with Cancer: A Systematic Review of Longitudinal Studies. Int. J. Environ. Res. Public Health.

[4] Obora M., Onsongo L., Ogutu J.O. (2022). Determinants of Sexual Function among Survivors of Gynaecological Cancers in a Tertiary Hospital: A Cross-Sectional Study. Ecancermedicalscience.

[5] Mohamad Muhit A.M., Sy-Cherng Woon L., Nik Mhd Nor N.S., Sidi H., Mohd Kalok A.H., Kampan N.C., Shafiee M.N. (2022). Sexual Dysfunction among Gynaecological Cancer Survivors: A Descriptive Cross-Sectional Study in Malaysia. Int. J. Environ. Res. Public Health.

[6] Takahashi M., Ohno S., Inoue H., Kataoka A., Yamaguchi H., Uchida Y., Oshima A., Abiru K., Ono K., Noguchi R. (2008). Impact of Breast Cancer Diagnosis and Treatment on Women’s Sexuality: A Survey of Japanese Patients. Psychooncology.

[7] Alananzeh I., Green H., Meedya S., Chan A., Chang H.C., Yan Z., Fernandez R. (2022). Sexual Activity and Cancer: A Systematic Review of Prevalence, Predictors and Information Needs among Female Arab Cancer Survivors. Eur. J. Cancer Care (Engl.).

[8] Taylor S., Harley C., Ziegler L., Brown J., Velikova G. (2011). Interventions for Sexual Problems Following Treatment for Breast Cancer: A Systematic Review. Breast Cancer Res. Treat..

[9] Emilee G., Ussher J.M., Perz J. (2010). Sexuality after Breast Cancer: A Review. Maturitas.

[10] Bartula I., Sherman K.A. (2015). The Female Sexual Functioning Index (FSFI): Evaluation of Acceptability, Reliability, and Validity in Women with Breast Cancer. Support. Care Cancer.

[11] Bartula I., Sherman K.A. (2015). Development and Validation of the Female Sexual Function Index Adaptation for Breast Cancer Patients (FSFI-BC). Breast Cancer Res. Treat..

[12] Karabulut Gul S., Tepetam H., Gursel O.K., Alanyali S., Oruc A.F., Tugrul F., Ergen S.A., Yavuz B.B., Kanyilmaz G., Altinok P. (2023). Investigating the Levels of Depression, Anxiety, Sexual Disorders, and Other Influencing Factors in Breast Cancer Patients: Turkish Radiation Oncology Integrative Group Study (TROD 12-05). Medicine.

[13] Carreira H., Williams R., Müller M., Harewood R., Stanway S., Bhaskaran K. (2018). Associations Between Breast Cancer Survivorship and Adverse Mental Health Outcomes: A Systematic Review. JNCI J. Natl. Cancer Inst..

[14] Flores N.J., Mathew M.J., Fortson L.S., Abernethy A.D., Ashing K.T. (2021). The Influence of Culture, Social, and Religious Support on Well-Being in Breast Cancer Survivorship. Cureus.

[15] Arceo-Martínez M.T., López-Meza J.E., Ochoa-Zarzosa A., Palomera-Sanchez Z. (2021). Estado Actual Del Cáncer de Mama En México: Principales Tipos y Factores de Riesgo. Gac. Mex. Oncol..

[16] Hietanen A.-E., Pick S. (2015). Gender Stereotypes, Sexuality, and Culture in Mexico. Psychology of Gender Through the Lens of Culture.

[17] Andrews C., Oths K., Dressler W. (2022). Age at Arrival and Depression among Mexican Immigrant Women in Alabama: The Moderating Role of Culture. Int. J. Environ. Res. Public Health.

[18] Lee Y., Lim M.C., Joo J., Park K., Lee S., Seo S., Lee D.O., Park S.-Y. (2014). Development and Validation of the Korean Version of the Female Sexual Function Index-6 (FSFI-6K). Yonsei Med. J..

[19] Guendler J.D.A., Amorim M.M., Flamini M.E.M., Delgado A., Lemos A., Katz L. (2023). Analysis of the Measurement Properties of the Female Sexual Function Index 6-Item Version (FSFI-6) in a Postpartum Brazilian Population. Rev. Bras. Ginecol. Obs./RBGO Gynecol. Obstet..

[20] Espitia De La Hoz F.J. (2018). Prevalencia y Caracterización de Las Disfunciones Sexuales En Mujeres, En 12 Ciudades Colombianas, 2009-2016. Rev. Colomb. Obs. Ginecol..

[21] Pais Ribeiro J.L., Martins da Silva A., Vilhena E., Moreira I., Santos E., Mendonça D. (2018). The Hospital Anxiety and Depression Scale, in Patients with Multiple Sclerosis. Neuropsychiatr. Dis. Treat..

[22] Salari N., Hasheminezhad R., Abdolmaleki A., Kiaei A., Shohaimi S., Akbari H., Nankali A., Mohammadi M. (2022). The Effects of Smoking on Female Sexual Dysfunction: A Systematic Review and Meta-Analysis. Arch. Womens Ment. Health.

[23] Di Francesco S., Caruso M., Robuffo I., Militello A., Toniato E. (2019). The Impact of Metabolic Syndrome and Its Components on Female Sexual Dysfunction: A Narrative Mini-Review. Curr. Urol..

[24] Wang X., Ji X. (2020). Sample Size Estimation in Clinical Research. Chest.

[25] Faul F., Erdfelder E., Buchner A., Lang A.-G. (2009). Statistical Power Analyses Using G*Power 3.1: Tests for Correlation and Regression Analyses. Behav. Res. Methods.

[26] Kang H. (2021). Sample Size Determination and Power Analysis Using the G*Power Software. J. Educ. Eval. Health Prof..

[27] Rosner B. (2011). Fundamentals of Biostatistics/Bernard Rosner.

[28] Levine M., Ensom M.H. (2001). Post Hoc Power Analysis: An Idea Whose Time Has Passed?. Pharmacotherapy.

[29] ClinCalc.com » Statistics » Post-hoc Power Calculator Post-Hoc Power Calculator. Evaluate Statistical Power of an Existing Study. https://clincalc.com/stats/Power.aspx.

[30] Pérez M., Liu Y., Schootman M., Aft R.L., Schechtman K.B., Gillanders W.E., Jeffe D.B. (2010). Changes in Sexual Problems over Time in Women with and without Early-Stage Breast Cancer. Menopause.

[31] Vitorino C.N., Omodei M.S., de Souza R.C., Nahas G.P., de Araujo Brito Buttros D., Carvalho-Pessoa E., Vespoli H.D.L., Nahas E.A.P. (2024). Assessment of Sexual Function in Postmenopausal Breast Cancer Survivors. Sex. Med..

[32] Valpey R., Kucherer S., Nguyen J. (2019). Sexual Dysfunction in Female Cancer Survivors: A Narrative Review. Gen. Hosp. Psychiatry.

[33] Del Pup L., Villa P., Amar I.D., Bottoni C., Scambia G. (2019). Approach to Sexual Dysfunction in Women with Cancer. Int. J. Gynecol. Cancer.

[34] Pitman A., Suleman S., Hyde N., Hodgkiss A. (2018). Depression and Anxiety in Patients with Cancer. BMJ.

[35] Fallowfield L., Jenkins V. (2014). Psychosocial/Survivorship Issues in Breast Cancer: Are We Doing Better?. JNCI J. Natl. Cancer Inst..

[36] Joaquim A., Amarelo A., Antunes P., Garcia C., Leão I., Vilela E., Teixeira M., Duarte B., Vieira M., Afreixo V. (2024). Effects of a Physical Exercise Program on Quality of Life and Physical Fitness of Breast Cancer Survivors: The MAMA_MOVE Gaia After Treatment Trial. Psychol. Health Med..

[37] Cooper M.L., O’Hara R.E., Martins J. (2016). Does Drinking Improve the Quality of Sexual Experience?: Sex-Specific Alcohol Expectancies and Subjective Experience on Drinking Versus Sober Sexual Occasions. AIDS Behav..

[38] Gutiérrez Zúñiga C., De La Torre Castellanos R. (2017). Census Data Is Never Enough: How to Make Visible the Religious Diversity in Mexico. Soc. Compass.

[39] Pepe F., Panella M., Pepe G., D’Agosta S., Pepe P. (1989). Frequency of Sexual Dysfunctions Among Roman Catholic Women. Fam. Pract..

[40] Mitchell J., Lannin D.R., Mathews H.F., Swanson M.S. (2002). Religious Beliefs and Breast Cancer Screening. J. Womens Health.

[41] Esfahani S.B., Pal S. (2018). Obesity, Mental Health, and Sexual Dysfunction: A Critical Review. Health Psychol. Open.

[42] Davis S.R., Lambrinoudaki I., Lumsden M., Mishra G.D., Pal L., Rees M., Santoro N., Simoncini T. (2015). Menopause. Nat. Rev. Dis. Primers.

[43] Cachelin F.M., Monreal T.K., Juarez L.C. (2006). Body Image and Size Perceptions of Mexican American Women. Body Image.

[44] Fingeret M.C., Teo I., Epner D.E. (2014). Managing Body Image Difficulties of Adult Cancer Patients: Lessons from Available Research. Cancer.

[45] Brederecke J., Heise A., Zimmermann T. (2021). Body Image in Patients with Different Types of Cancer. PLoS ONE.

[46] Donkers H., Smits A., Eleuteri A., Bekkers R., Massuger L., Galaal K. (2019). Body Mass Index and Sexual Function in Women with Gynaecological Cancer. Psychooncology.

[47] Nelson B. (2011). The “Cancer of Machismo” in Mexico. Cancer Cytopathol..

